# Comparison of Donepezil, Memantine, Melatonin, and Liuwei Dihuang Decoction on Behavioral and Immune Endocrine Responses of Aged Senescence-Accelerated Mouse Resistant 1 Mice

**DOI:** 10.3389/fphar.2020.00350

**Published:** 2020-04-28

**Authors:** Ju Zeng, Xiaorui Zhang, Jianhui Wang, Xiaorui Cheng, Yongxiang Zhang, Wenxia Zhou

**Affiliations:** ^1^Department of Neuroimmunopharmacology, Beijing Institute of Pharmacology and Toxicology, Beijing, China; ^2^State Key Laboratory of Toxicology and Medical Countermeasures, Beijing, China

**Keywords:** aging, Liuwei Dihuang decoction, cognition, immune response, inflammation

## Abstract

Aging is a natural biological process associated with cognitive decline and neuroendocrine–immune system changes; the neuroendocrine–immune system plays crucial role in brain aging and neurodegeneration, and it is essential to discern beneficial attempts to delay the aging progress based on immunological aging. In this study, we have investigated the effects of Traditional Chinese Medicine (TCM)—Liuwei Dihuang decoction (LW)—and donepezil, memantine, and melatonin on cognitive decline in aging mice. The aged SAMR1 mice received oral administration of donepezil (1mg/kg), memantine (10 mg/kg), melatonin (10 mg/kg), and LW (10 g/kg) for 3 months. A shuttle box, Morris water maze, and elevated-zero maze were performed to assess cognitive function, and flowcytometry, Luminex, and radioimmunoassay were performed to measure the lymphocyte subsets, inflammatory factors, and hormones. We observed that survival days of mice was increased with melatonin and LW, the anxiety behavior was significantly improved by memantine, melatonin, and LW treatment, active avoidance responses significantly improved by LW, donepezil, and memantine, the spatial learning ability was significantly improved by donepezil, and LW and melatonin were beneficial to the spatial memory of old mice. For immune function, LW increased CD4^+^ and CD4^+^CD28^+^ cells and reduced TNF-α, IL-1β, and G-CSF in plasma, and it also promoted the secretion of anti-inflammatory factors IL-4, IL-5, and IL-10 by regulating the active of Th2 cells in spleen. Donepezil and memantine exerted protective effects against CD4^+^CD28^+^ cell decrease caused by aging and reduced the pro-inflammatory factors TNF-α, IL-1β, and G-CSF in plasma. Melatonin could reverse CD8^+^CD28^+^ cell imbalances and increased B cells. For endocrine factors, LW increased TSH levels in the pituitary, and melatonin increased the GH level in blood. Our findings indicated that LW improved the cognitive decline in aging mice, and this might be associated with modulation of the active T cells and HPG axis hormones as well as increasing anti-inflammatory factors. Meanwhile, donepezil and memantine have advantages in regulating adaptive immunity, melatonin has advantages in the regulation of B cells and pituitary hormones, and LW exhibits a better effect on neuroendocrine immune function compared with the others from a holistic point of view. LW might be a potential therapeutic strategy for anti-aging-related syndromes, and it can also provide a value on medication guidance about drug combinations or treatment in clinic.

## Introduction

Aging is a natural and an irreversible process of life that is associated with cognitive decline. It has been noted that cognitive performance, such as attention, learning, working, episodic memory, processing speed, etc. obviously declined among older people (Levy, 1994; Salthouse, 2010a; Harada et al., 2013), and immune system changes that develop with increasing age, referred to as immunosenescence, have shown that aging can affect both the innate and adaptive immune response. The changes in soluble immunological mediators with aging are descripted as “inflammaging” in literature, and this is defined as a low-grade persistent increase in inflammatory molecules. (Aw et al., 2010; Gruver et al., 2007). The clinical consequences of the aging immune system disorder include susceptibility to infection and malignancy, and these are important in the process of neurodegenerative diseases. Aging is a major risk factor for neurodegenerative diseases, the most common being Alzheimer’s disease (AD), but these also include Parkinson disease (PD), amyotrophic lateral sclerosis (ALS), and so on (Petersen et al., 1997; Moraros et al., 2017). Due to advances in health care, the resulting increase in the life expectancy of the population, as well as the lack of an effective treatment, the prevalence of neurodegenerative diseases is increasing and imposing a significant burden on the aging population. It is consequently important to study the mechanism and therapeutic strategies for delaying senescence. Evidence has shown that age-related cognitive decline is associated with oxidative stress, inflammatory responses, and innate and adaptive immune disorders, and it is also subjected to the effects of stress hormones and alterations of the hypothalamic-pituitary axis (HPA) function. Compared to young mice, the response and proliferation of CD4^+^ and CD8^+^cells were reduced, the expression of CD28^-^ was elevated, the levels of some inflammatory factors were elevated (TNFα, IL-1β etc.), corticosterone increased, and growth hormone decreased in aging mice. There are some kinds of therapeutic strategies for the improvement of disease- and aging-related cognitive decline; currently, the main areas of the development of pharmacological treatments targeting age-related functional decline and pathological manifestation are focused on some important aging-related genes, such as Sir2 and TOR pathway genes (Blagosklonny, 2007; Vaiserman et al., 2016), and most of these genes represent promising drug targets. On the other hand, an evaluation of the pharmacological potential of drugs that has been approved by the Food and Drug Administration (FDA) for the treatment of particular pathological conditions related to aging has commenced (Vaiserman et al., 2016). Rapamycin (Wilkinson et al., 2012; Neff et al., 2013), metformin (Anisimov et al., 2010; Cabreiro et al., 2013), resveratrol (Kaeberlein, 2010; Park et al., 2012), and some anti-inflammatory drugs are among these medications, and the evidence has also proven that melatonin could delay senescence *via* anti-oxidative stress. Additionally, memantine and donepezil are the effective drugs for alleviating the progress of AD in clinic, but the preparations of these drugs are commonly used to treat patients with particular chronic diseases, and they have not been used/evaluated for the pharmacological potential of aging-related decline in cognitive and physiological functions, especially immune and endocrine functions, in the absence of clinical manifestations of particular diseases. However, the immune and endocrine system have crucial functions in brain aging and neurodegeneration, and, when studying age-related neurodegenerative diseases in animal models, it is thus necessary to consider aging. Immunological aging includes changes in the adaptive immune system, inflammation response, and disruptions in the hormone axis, specifically altered cortisol levels and glucocorticoid signaling (Reed, 2019), and evidence has indicated than growth hormone deficits have been linked to deficits in memory (Sonntag et al., 2005). The hypothalamus might have a role in regulating organismal aging (Zhang et al., 2013). It will be essential to discern beneficial attempts to delay the aging progress from the point of view of immunological aging. Studying the effects of the drugs on the immune and endocrine systems would likely provide a strategy for preventing or treating specific conditions that are associated with aging in the performance of anti-aging drugs.

Traditional Chinese Medicine (TCM) has a solid theoretical foundation and experience in disease preservation. It has the characteristics of being able to tackle multiple targets, mild efficacy, and fewer side effects. Liuwei Dihuang decoction (LW) is a classical TCM that is comprised of *Radix Rehmanniae, Rhizoma dioscoreae, Fructus corni., Cortex moutanradicis, Rhizoma Alismatis, Paeonia suffru-ticosa Andrews*, and *Poria*. It has a long usage history in the prevention and treatment of diverse diseases, including type-2 diabetes (Hsu et al., 2014), hypertension (Der-Shiang et al., 2014), and improving cognitive ability impairment caused by disease. It has been reported that LW could promote the repair of the dentate gyrus (Lee et al., 2005), restoring the cholinergic system of the central nervous system (Zhang et al., 2011), has an anti-ovarian aging in D-galactose-induced rats (Wang et al., 2013), and exhibits a protective effect on dopaminergic neurons by enhancing antioxidant defense and reducing apoptotic death (Tseng et al., 2014). Additionally, LW extract could alleviate the toxicity induced by the β-amyloid protein (Sangha et al., 2012), and it could regulate the hypothalamus–pituitary–ovarian (HPO) axis functional interference in senescence-accelerated mouse prone 8 strain (SAMP8) (Zhang et al., 2004). However, there are few studies on natural normal aging model mice, especially SAMR1, which were used as the controls for the SAMP8 mice. This made us consider whether donepezil, memantine, and melatonin could improve cognitive function in SAMR1, and the underlying mechanisms of LW in normal aging-related cognitive decline are not clear. Senescence-accelerated mouse resistant 1 strain (SAMR1) mice were developed from AKR/J mice based on a graded score for senescence and pathologic phenotypes, and this strain meets most criteria for the use of mammalian models for aging research, such as life table data, short life span, defined environmental conditions, genetic characteristics, etc. (Takeda et al., 1997a; Takeda et al., 1997b). SAMR1 mice showed a normal aging process, and had more obvious characteristics of aging-related performance, such as hair loss, glossiness, and reactivity, and they also exhibited age-related behavior changes (Umezawa et al., 1999). This mouse strain is a valid strain for aging research and is a good model for studying the balance of the neuroendocrine–immune regulatory network (Takeda et al., 1997). In this study, we investigated the effects of donepezil, memantine, melatonin, and LW on cognitive decline in normal aging mice SAMR1, and we also analyzed the effects on endocrine and immune functions. This should provide promising therapeutic strategies for delaying aging and improving cognitive function from the view of immune endocrine function.

## Materials and Methods

### Animal

SAMR1 were kindly provided by Dr. T. Takeda (Kyoto University, Japan) (Takeda et al., 1997a), and bred in our institute. In this study, 19~21month SAMR1 mice were used as subjects. All of the animals were housed on a 12 h light–dark cycle at 23 ± 1 °C and 50 ± 5% humidity in groups of five in plastic cages with corn cob bedding. Pellet food (provided by the animal center of the AMMS) and water were delivered ad libitum. Mice were acclimatized to the laboratory environment for at least one week prior to the experiment. The animals received humane care according to the National Institutes of Health (USA) guidelines approved by the Institute of Animal Care and Use Committee (IACUC) of the National Beijing Center for Drug Safety Evaluation and Research (NBCDSER) (No. 2018-030).

### Drug Preparation and Administration

**Figure 1:**
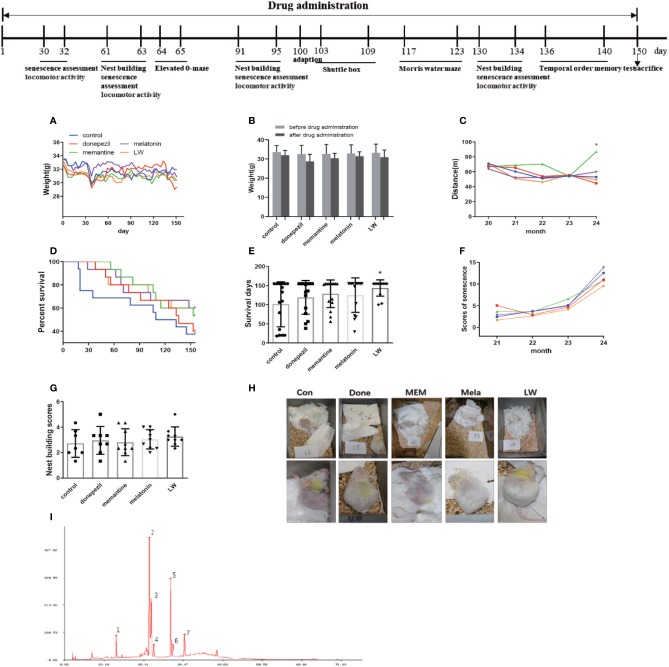
The experimental timeline and the effects of donepezil, memantine, melatonin, and LW on the aging performance of old SAMR1 mice. **(A)** The weight of mice during drug administration. **(B)** The locomotor activity of mice during drug administration. **(C)** The score of mouse nest building after 3 months of treatment. **(D)** The percentage survival of the mice. Log-rank test of survival curve, P=0.1. **(E)** Survival days of mice. **(F)** The aging degree score of mice during drug administration. One-way ANOVA and Dunnett test, mean ± S.D., n=13~16 (male: n = 4~6, female: n = 7~9). **(G)** The nest building scores. **(H)** Performance of nest building and fur of mice. **(I)** The peak of 1, 2, 3, 4, 5, and 7 are the main compounds α-morroniside, β-morroniside, oxypaeoniflorin, loganin, paeoniflorin, and 5-hydroxymethylfurfural of LW, respectively.

The origin herbs of Liuwei Dihuang (LW) were purchase from Beijing Tongrentang Co., Ltd., and they were comprised of *Radix Rehmanniae* (Lot.20150130)*, Rhizoma dioscoreae* (Lot. 1503028)*, Fructus corni*.(Lot.20151126)*, Cortex moutanradicis* (Lot.20160526)*, Rhizoma Alismatis* (Lot.20160116)*, Paeonia suffru-ticosa Andrews* (Lot.20151126), and *Poria* (Lot.1601001) at a weight ratio of 8:4:4:3:3:3. The herbs were extracted twice by six times water (volume/weight) for 1.5 h each time. Then, the extracted solution was filtered and mixed together, and it was then concentrated to a relative density of 1g/ml for use, which is equivalent of the clinical dosage for humans. The high-performance liquid chromatography (HPLC) characteristic chromatogram of LW was previously established and used for its quality control (Zhang and Zhao, 2006), and the voucher specimens were deposited at the Beijing Institute of Pharmacology and Toxicology. The main compounds, α-morroniside, β-morroniside, oxypaeoniflorin, loganin, paeoniflorin, and 5-hydroxymethylfurfural of LW, were detected by HPLC (**Figure 1I**). Donepezil (Lot#7FH2N-JS, CAS:120014-06-4, MW: 379.492g/mol, ≥98%; Beijing Ouhe Tech Co.), Memantine (Lot#150825, CAS: 41100-52-1, MW: 179.3g/mol, > 98%; Sinopharm Chemical Reagent Co., Ltd), Melatonin (CAS: 73-31-4, MW: 232.28g/mol, M5250, sigma). The experiments were performed in 19–21-month-old SAMR1 mice (n =76, male: 30, female: 46), which randomly divided into five groups: the control group had 17 mice (six male and 10 female), and four drug treatment groups had 16 mice in each group (six male and nine female). The drug treatment groups were given intragastric administration of donepezil (1mg/kg), memantine(10mg/kg), melatonin(10mg/kg), or LW for 3 months, and they then took a series of behavioral test. During this period, the drug administration continued until the mice were sacrificed (150 total days). The mice were weighed every 3 days, and the locomotor activity and degree of senescence were evaluated every 30 days.

### Evaluation of Senescence and Nest Building

The degree of senescence in SAMR1 mice were evaluated by a grading score system developed by the Hosokawa group (Hosokawa et al., 1984). Briefly, according the senescence process, the score system selected 11 categories from the clinical signs and gross lesions considered to be closely associated with aging. The grade ranged from 0 to 4, and it was designed to assess changes in the appearance of the mice, including reactivity, passivity, hair loss, glossiness, coarseness, ulcer, periophthalmic lesions, cataracts, corneal ulcers, corneal opacity, and lordoscoliosis. Grade 0 represented no particular change, and grade 4 represented the most severe changes. Details refer to reference Hosokawa et al. (1984). We chose napkin as the material for nest building performance. Nest quality is quantified with complexity scores of 1–5 grades followed by Deacon’s method and scoring criteria (Deacon, 2006): 1 score: did not see and move; 2 scores: partially shredded; 3 scores: most torn but no clear direction; 4 scores: a definite direction but smooth; 5 scores: a complete nest with walls surrounding the mice.

### Elevated Zero Maze Test

The elevated zero maze is an improvement upon the elevated plus maze, which saves time spent in the traditional “plus” design to connect the central circle, and it also allows for continuous detection (Shepherd et al., 1994; Kulkarni et al., 2007). The maze is an annular dark gray platform (60cm in diameter) constructed of plexiglas divided into four equal quadrants. Two opposite quadrants were “open arm,” and the remaining two “closed arm” quadrants were surrounded by 16cm-high dark gray walls (Shanghai xinruan Information Technology Co., Ltd., shanghai, China). Mice were placed in the test room to adapt to the environment for 30min. To start the test, each mouse was placed at a randomly chosen boundary between an open and a closed zone, facing the inside of the closed arm, and each mouse explored for 5min freely. The indicators recorded using Super Maze animal behavior video analysis software, and they including opening and closing area residence time, entering the opening and closing area times, and movement distance.

### Morris Water Maze Test

The Morris water maze consisted of a large circular pool (100cm diameter, 45cm high) with a black inner surfer that was placed in a dimly lit, soundproofed room with various visual cues. The pool was divided into four quadrants, and a black platform (8cm in diameter) was placed 1.0 cm below the water level in the middle of a fixed quadrant. The water was adjusted to 20 ± 1°C. The task consisted of a training and probe test to assess spatial learning and memory. The training was performed over four trials daily for six consecutive days. The mice were placed at four different start points into the water facing the wall of the pool to swim freely to seek the hidden platform. The escape latency was defined as the time spent seeking the platform and whether or not they stayed on it for at least 10s; if they did not find the platform within 60s, they were placed onto the platform for 10s. After 24h of the last trial to assess memory retention, the platform was removed, and the mice were allowed to carry out a single probe test to swim for 60s in the maze to find the position of the platform. During the whole task, the mouse performance was recorded with a camera located at the ceiling and analyzed by video-tracking system of ANY-maze software.

### Shuttle Box Test

The apparatus consisted of two connected boxes (266mm×160mm×200mm) with a gate, and there was a buzzer at the top and an electric stimulus grid at the bottom (Shanghai Xinruan Information Technology Co., Ltd., shanghai, China). The procedure of the shuttle box was carried according to previous study (Cheng et al., 2011). Briefly, before the test, we allowed the mice to freely move in the chambers for 3min to adapt to the environment. This was then followed by 30 trails with a 30s inter-trial interval. They were given conditional stimulus (sound and light) for 10s followed by 3s unconditional stimulation an electrical foot shock (0.3mA), and this training session was performed for 6 days. According to each point of 10 trainings, the active avoidances were recorded as the mice escaped to the other chamber in the period of conditional stimulus, and the ratio of the number of active avoidances to the total number was used as an index reflecting the learning effect of mice. On the 7th day, the retention session was also followed by 30 trails without foot shock, and the number of active avoidances was recorded to reflect the memory ability. In order to avoid fatigue interfering with this test, we gave the mice one week to recover after the Morris water maze test before we then carried out the shuttle box test.

### Tissue Preparation

After completion of all behavior tests, the mice were sacrificed: we removed the brains, the hemisphere was stored at −80°C until used, the other hemisphere was immersed in 4% paraformaldehyde dissolved in phosphate-buffered saline (PBS) (pH 7.4), and we then fixed these in 10% buffered formalin, embedded them using paraffin, and prepared them for immunofluorescence staining procedures. The blood was dropped into a tube with an anticoagulant placed at 4°C for 2 h, and it was then centrifugated at 3000g for 15min. The plasma was collected into clean tubes and stored at -70°C for measurements.

### Cytokine Measurement

To measure the cytokine changes in the plasma samples, the levels of granulocyte colony stimulating factor (G-CSF), monocyte chemotactic protein (MCP)-1, tumor necrosis factor (TNF)-α, interferon (IFN)-γ, interleukin (IL)-1β, IL-4, IL-5, IL-6, and IL-10 were detected using a multiplex map kit (MCYTOMAG-70K, Millipore). The procedure was followed using the instructions and analyzed using Luminex 200™ (Luminex, Austin, TX, USA).

### Flow Cytometry

To acquire single-cell suspension, the mouse spleen was minced through a 40µm nylon cell strainer, and we used the Tris-NH_4_Cl lysis buffer (0.017 M Tris-HCl, 0.144 M NH4Cl) to deplete the red blood cells. Then the spleen cells were harvested and divided into two tubes, and 100μL blood was dripped into a tube for testing. One tube of the sample was incubated with the antibody FITC anti-mouse CD3, APC anti-mouse CD4, PerCP anti-mouse CD8, PE anti-mouse CD28, and the other tube of cells were incubated with APC anti-mouse CD4, FITC anti-mouse CD25, and PerCP anti-mouse CD19 (BioLegend) at room temperature for 30 min in the dark before being washed and resuspended in 0.5 mL of PBS and quantified by flow cytometry (BD Calibur™, USA).

### Radioimmunoassay and ELISA of Hormones

To detect the hormone levels in the plasma, hypothalamuses, and pituitaries of the old mice, the tissues were weighed and boiled in 0.4 mL saline for 5 min, and the peptides were then extracted by homogenizing the tissues in 0.2 mL of 1M glacial acetic acid and 0.2 mL of 1M NaOH followed by centrifuging the mixture at 3000 rpm for 30 min. Supernatants were stored at -20°C. Concentrations of corticosterone, growth hormone (GH), estrogens (E2), and testosterone (T) were tested by radioimmunoassay with a ^125^I-corticosterone RIA kit, ^125^I- GH RIA kit, ^125^I- estrogens RIA kit, and a ^125^I- testosterone RIA kit (North Institute of Biological Technology, Beijing, China), and dehydroepiandrosterone (DHEA) was tested by ELISA with a DHEA-kit (Shanghailengton Biotechnology co., Ltd. China) in plasma. Concentrations of adrenocorticotropic hormone (ACTH), and Thyroid Stimulating Hormone (TSH) in supernatants extracted from pituitaries were determined with a ^125^I-ACTH RIA kit and ^125^I-TSH RIA kit (North Institute of Biological Technology, Beijing, China).

### Statistical Analysis

Data are presented as means ± standard errors. Statistical significance between groups was determined using a one-way Analysis of variance (ANOVA) and Kruskal-Wallis test by Graphpad prism 6.0 (Inc., La Jolla, CA, USA). The Correlation are analysis by Pearson correlation analysis. A probability value of less than 0.05 was considered a significant difference.

## Results

### The Effects of Donepezil, Memantine, Melatonin, and LW on Delaying the Aging Process of Old SAMR1 Mice

As we known, the decrease of basic activity ability is an important feature of aging. In order to investigate the effects of donepezil, memantine, melatonin, and LW on aging appearance, we detected the survival, weight, nest building, the degree of senescence, and locomotor activity of SAMR1 mice during the study. The results showed that the survival days of SAMR1 mice was significantly increased with LW treatment (**Figure 1E**), and an analysis of the survival curve indicated that chronic administration of melatonin and LW might improve the survival rate in the elderly (**Figure 1D**). The nest building was also better with LW treatment than with the control group, but this was without significant statistical difference (**Figure 1G, H**). Moreover, the locomotor activity in old SAMR1 mice with memantine treatment was increased compared with the control group (**Figure 1C**). There was no significant change in body weight with drug treatment, but the difference within the group showed that the weight loss of the donepezil group (3.8g) of mice was slightly higher than other groups (1.5–2.1g). (**Figures 1A, B**). There was no significant difference in overall evaluation of aging degree of mice (**Figure 1F**) during drug administration, but, from the appearance of mice in pictures, we could see that the mice treated with LW and melatonin were significantly better than those treated with donepezil and memantine. Additionally, **Table 1** gives the number of mice in each group in every behavioral test and the final survival of mice.

**Table 1:** The number of mice in every behavioral test and the final survival mice.

Group	Total (n)	Final (survival/n)	Survival ratio	The number and age of mice in behavioral test					
				Average age	Elevated-0 maze	Average age	Shuttle box	Average age	MWM
Control	16, M: 6, F: 10	6, M: 2/6, F: 4/10	37.5%	22 mo.	12 (M: 4, F: 8)	23 mo.	9 (M: 4, F: 5)	23.5 mo.	7 (M: 2, F: 5)
Donepezil	15, M:6, F: 9	6, M: 2/6, F: 4/9	40%	22 mo.	12 (M: 5, F: 7)	23 mo.	10 (M: 4, F: 6)	23.5 mo.	7 (M: 2, F: 5)
Memantine	15, M: 6 F:9	6, M: 3/6, F: 5/9	53.3%	22 mo.	13 (M: 5, F: 8)	23 mo.	10 (M: 4, F: 6)	23.5 mo.	8 (M: 3, F: 5)
Melatonin	15, M: 6, F: 9	9, M: 4/6, F: 5/9	60%	22 mo.	11 (M: 5, F: 6)	23 mo.	10 (M: 4, F: 6)	23.5 mo.	8 (M: 3, F: 5)
LW	15, M: 6, F: 9	9, M: 3/6, F: 6/9	60%	22 mo.	12 (M: 5, F: 7)	23 mo.	10 (M: 4, F: 6)	23.5 mo.	8 (M: 4, F: 4)

M, male; F, female.

### The Effects of Donepezil, Memantine, Melatonin, and LW on the Anxiety-Like Behavior in Old SAMR1 Mice

**Figure 2:**
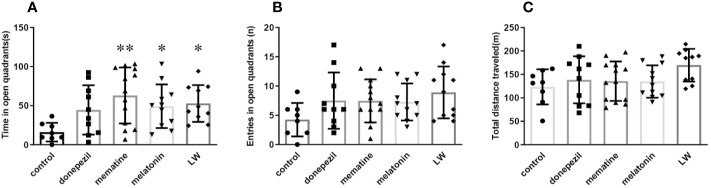
Effects of donepezil, memantine, melatonin, and LW on anxiety behavior in old SAMR1 mice. **(A)** Time spent in the open quadrants, **(B)** number of the open quadrants entries, and **(C)** total distance traveled in the elevated zero maze test. *P < 0.05, **P < 0.01 *vs* control group. Kruskal-Wallis test, mean ± S.D., n=11~13(male: n = 4~5, female: n = 7~8).

Aged rats/mice were much more affected by anxiety compared to the young ones, and an elevated zero maze was used to measure the anxiety-like behavior, and it could also create anxiety in aged mice (Küçük et al., 2008; Shoji and Mizoguchi, 2010). In order to know whether donepezil, memantine, melatonin, and LW had the effect of regulating the emotions of aging mice, we chose the elevated zero maze to evaluate the anxiety behavior in the aging mice. The results showed that administrations with memantine, melatonin, and LW for 2 months could significantly improve the exploration time of mice in the open regions (*P* < 0.05) (**Figure 2A**), and LW showed better improvement tendencices in entries in open quadrants and the total distance traveled in the maze (**Figures 2B, C**) as well. The data indicated that long-time administration of LW has an anxiolytic property in aging mice.

### Improvement of Active Avoidance Ability and Spatial Memory Decline Associated With Aging by Donepezil, Memantine, Melatonin, and LW Administration

**Figure 3:**
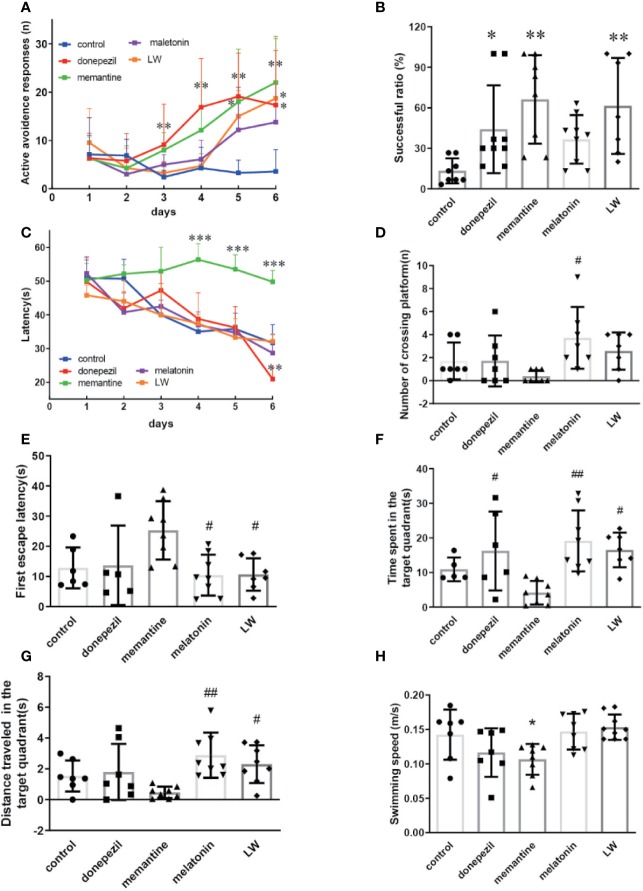
The effects of donepezil, memantine, melatonin, and LW on the active avoidance responses, spatial learning and memory in old SAMR1 mice. The successful avoidance times in training phase **(A)** and testing phase **(B)** in shuttle box test. n=9~10(male: n=4, female: n=5~6).The escape latency of mice in learning task **(C)** and probe trial **(E)**, number of crossing the platform **(D)**, the time spent in the target quadrant **(F)**, distance traveled in the target quadrant **(G)**, and swimming speed **(H)** in the Morris water maze test; *P < 0.05, **P < 0.01, ***P < 0.001, *vs* control group. Kruskal-Wallis test. ^#^*P* < 0.05, ^##^*P* < 0.01, *vs* memantine group, Kruskal-Wallis test. mean ± S.D., n = 7~9 (male: n = 2~4, female: n=5).

Based on the observation that aging lead to extensions in avoidance responses and also impairs spatial learning (Küçük et al., 2008), the Morris water maze and shuttle box test were chosen to evaluate the hippocampus-dependent memory functions of old SAMR1 mice. The results showed that the active avoidance ability of old SAMR1 mice was poorer. After 3 months of drug administration, LW, memantine, and donepezil significantly increased (*P* < 0.01) the number of active avoidance responses compared with the control group at the 6^th^ training day (**Figure 3A**); in the test session, the number of active avoidance responses in old SAMR1 mice were significantly increased (*P* < 0.01) with LW, memantine, and donepezil administration (**Figure 3B**). In the MWM task, the donepezil treatment significantly decreased the escape latency of mice at the last training day (*P* < 0.05) (**Figure 3C**); in probe test, the number of crossing platform exhibited a notable upward trend by LW and melatonin administration (**Figure 3D**), the first escape latency to reach the platform of LW and melatonin were significantly shorter than others (**Figure 3E**) and the time spent (**Figure 3F**) and distance traveled (**Figure 3G**) in the target quadrant also increased with LW and melatonin treatment compare with control group and showed significant improvement when compared to other drugs. Interestingly, the mice administrated with memantine showed better active avoidance responses than other drugs group in shuttle box task, but they had worse performance in MWM task, and the swimming speed (**Figure 3H**) was significantly decreased compared to the control group. These data indicated that long-term administration of LW, memantine, and donepezil could improve the active avoidance responses of aged mice, while LW and melatonin are beneficial to the spatial memory of aged mice. Furthermore, we also investigated the object discrimination behaviors of aged SAMR1 mice by temporal order memory test, and the results showed that memantine, melatonin, and LW exhibited tendencies to improve the temporal order memory in old SAMR1 mice (**Figure S1**). We comprehensively observed the performances of these four drugs, and they indicated that LW may have a more comprehensive effect, not only the improvement of the active avoidance response but also in its upward tendency for improving aging-related memory disorders.

### The Modulation of Lymphocyte Subsets in the Spleen of Old SAMR1 Mice by Donepezil, Memantine, Melatonin, and LW Administration

**Figure 4:**
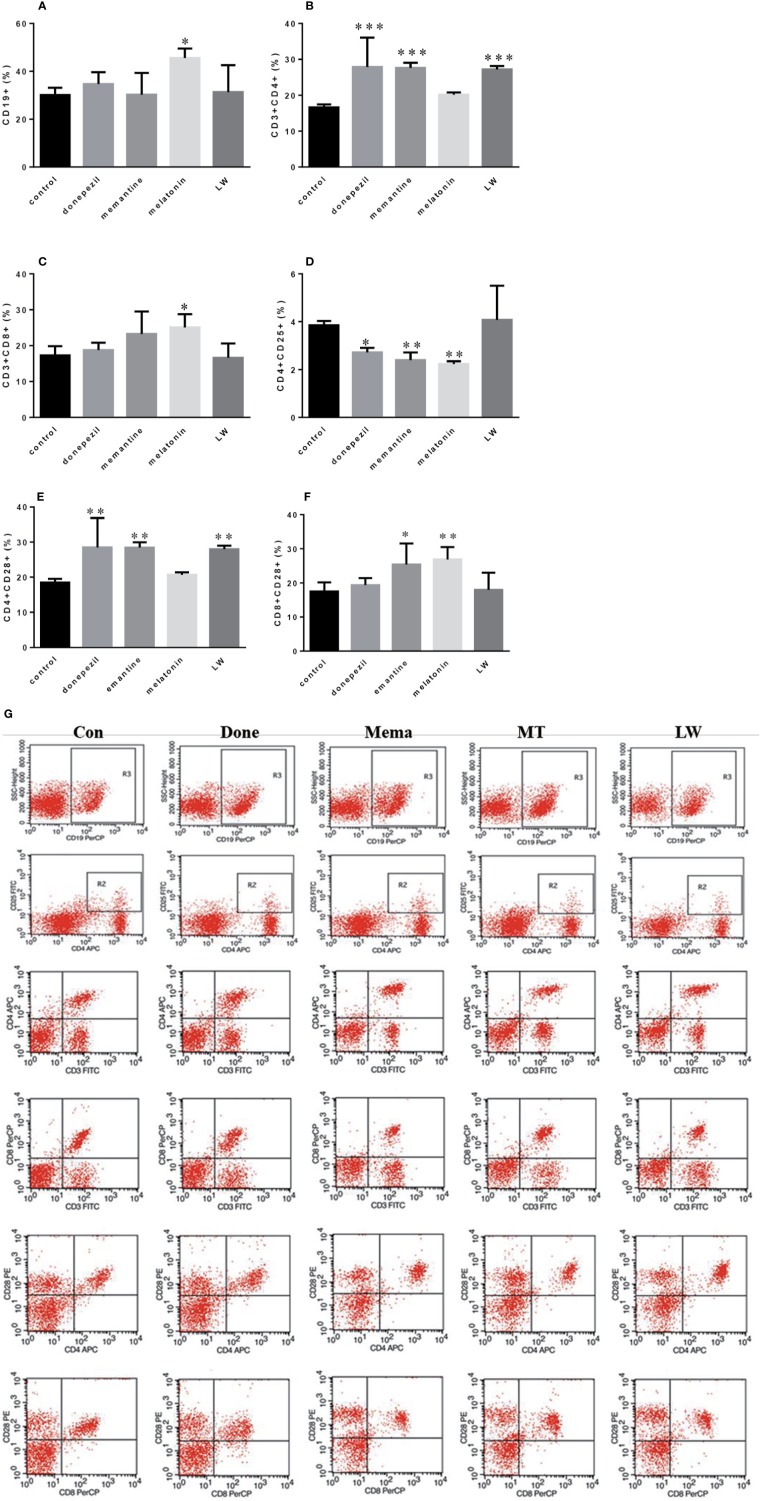
Effects of donepezil, memantine, melatonin, and LW on lymphocyte subsets of splenocyte in old SAMR1 mice. 1× 10^6^ spleen cells isolated from mouse (n = 6) spleen were harvested, **(A)** CD19^+^ B cells, **(B)** CD3^+^ CD4^+^ T cells, **(C)** CD3^+^ CD8^+^ T cells, **(D)** CD4^+^ CD25^+^ T cells, **(E)** CD4^+^ CD28^+^ T cells, **(F)** CD8^+^ CD28^+^ T cells quantified by flow cytometry. *P < 0.05, **P < 0.01, ***P < 0.001 *vs* control, one-way ANOVA and Dunnett test. mean ± S.D., n=6~9(male: n = 2~4, female: n = 4~6). **(G)** The scatter plot of lymphocyte subsets.

In order to investigate the effects of drugs on immune changes in aging SAMR1 mice, we analyzed spleen lymphocytes in aging SAMR1 mice. The B lymphocyte CD19^+^ cell was significantly increased (*P* < 0.05, F=4.183) in melatonin-administrated mice compared with the control group (**Figure 4A**). The ratio of T lymphocyte subsets CD3^+^CD4^+^T cell (*P* < 0.001, F=9.605) and CD4^+^CD28^+^T cell (*P* < 0.001, F=8.653) was significantly increased with LW, donepezil, and memantine administration (**Figures 4B, E**); the CD3^+^CD8^+^T cell (*P* < 0.05, F=3.757) (**Figure 4C**) was only increased with melatonin treatment, and the CD8^+^CD28^+^T cell (*P* < 0.05, F=4.722) was increased with memantine and melatonin treatment (**Figure 4F**). The CD4^+^CD25^+^T cell was significantly decreased following donepezil (*P* < 0.05, F=8.390), memantine, and melatonin (*P* < 0.01, F=8.390) administration (**Figure 4D**). And scatter plot as shown in the **Figure 4G**. Additionally, we also detected lymphocytes in peripheral blood, found the regulation of lymphoid subsets in spleen was stronger than in blood after drug treatment, and that there was only the CD3^+^CD8^+^T cell was significantly upregulated in memantine- (*P* < 0.05, F=6.027), melatonin- (*P* < 0.001, F=6.027), and LW-treated mice (*P* < 0.01, F=6.027) (**Figure S2**). These results indicated that LW, donepezil, and memantine could improve the T lymphocyte subsets proliferation and response of aging SAMR1 mice.

### Regulation of the Abnormal Secretion of Cytokines in Old SAMR1 Mice by Donepezil, Memantine, Melatonin, and LW Administration

**Figure 5:**
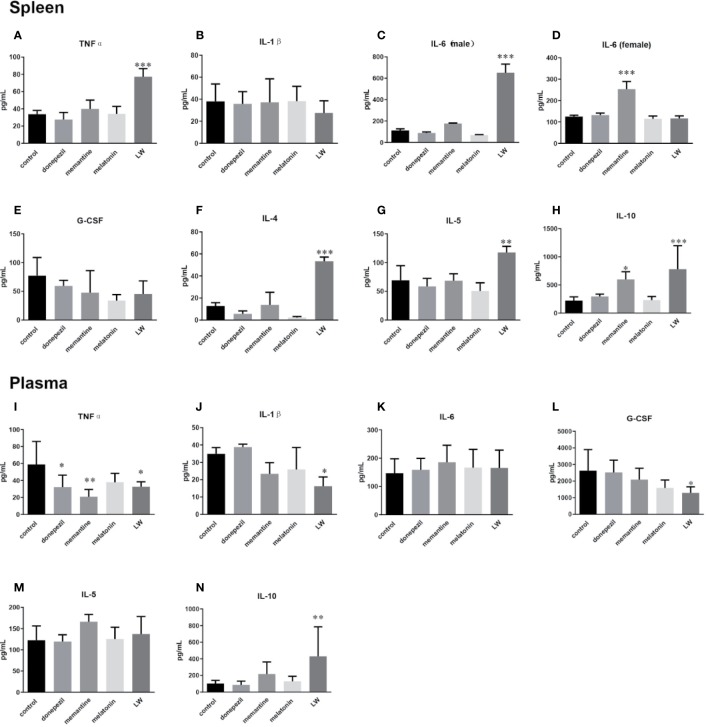
Effects of donepezil, memantine, melatonin, and LW on cytokine levels in the splenocyte culture supernatant and plasma of old SAMR1 mice. After the behavior testes, the mice were sacrificed, and spleen cells isolated from the spleen were harvested, 5×10^5^ cell/mL was seeded in 96-well plates, cultured for 56 h, and then the supernatants were harvested for cytokine detection. **(A, I)** tumor necrosis factor α (TNF-α), **(B, J)** interleukin-1β (IL-1β), **(C, D, K)** interleukin-6 (IL-6), **(E, L)** granulocyte colony stimulating factor (G-CSF), **(F)** interleukin-4 (IL-4), **(G, M)** interleukin-5 (IL-5), **(H, N)** interleukin-10 (IL-10). *P < 0.05, **P < 0.01, ***P < 0.001, *vs* control, one-way ANOVA and Dunnett test, Kruskal-Wallis test. mean ± S.D., *n* = 6~9(male: n=2~4, female: n=4~6).

To examine the effects of these drugs on the inflammatory response in aging mice, we detected some pro- and anti-inflammatory factors or chemokines in plasma and splenocyte culture supernatant. The results showed that in splenocyte culture supernatant, the levels of TNFα (*P* < 0.001) (**Figure 5A**), IL-4 (*P* < 0.001, F=35.8) (**Figure 5F**), IL-5 (*P* < 0.01, F=8.726) (**Figure 5G**), and IL-10 (*P* < 0.001, F=8.679) (**Figure 5H**) were significantly increased by LW treatment compared with control group, and IL-10 (*P* < 0.05, F=8.679) was also increased by memantine treatment. Notably, the regulation of LW on IL-6 in the spleen of old female and male mice was different, and the level of IL-6 (**Figure 5C**) in male mice (*P* < 0.001, F=119.3) significantly increased while exhibiting no change in female mice, and memantine (*P* < 0.001, F=30.39) exhibited the opposite effect (**Figure 5D**). Besides, the secretion of IL-1β (**Figure 5B**) and G-CSF (**Figure 5E**) showed a downward trend in the LW treatment group. In plasma, the pro-inflammatory factors level of TNFα (*P* < 0.05, F=4.052) (**Figure 5I**) was significantly decreased with donepezil, memantine and LW treatment, the levels of IL-1β (**Figure 5J**), G-CSF (*P* < 0.05, F=3.754) (**Figure 5L**) were significantly decreased with LW treatment, and anti-inflammatory factor IL-10 (*P* < 0.01) (**Figure 5N**) was significantly elevated in old SAMR1 mice after LW administration. While IL-6 and IL-5 have no significant changes in all group (**Figure 5K, M**). These data demonstrate that LW has a good effect on the regulation of the imbalance level of Th1 and Th2 factors in aging mice, and it is better than melatonin, memantine, or donepezil from a systematic perspective.

### The Regulatory Effect on Hormone Secretion in Old SAMR1 Mice by Donepezil, Memantine, Melatonin, and LW Administration

**Figure 6:**
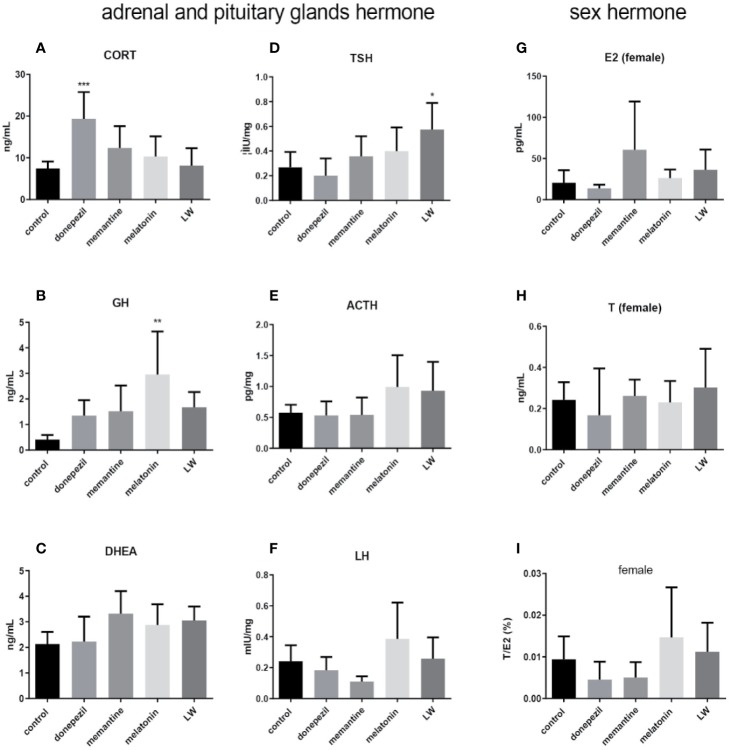
The neuroendocrine hormone secretion in old SAMR1 mice after donepezil, memantine, melatonin, and LW administration. **(A)** corticosterone (CORT), **(B)** growth hormone (GH), **(C)** dehydroepiandrosterone (DHEA) in the plasma, **(D)** thyrotropin (TSH), **(E)** adrenocorticotropic hormone (ACTH), **(F)** luteinizing hormone (LH) in the pituitary, **(G)** estradiol (E2), **(H)** testosterone (T), **(I)** the ratio of T/E2 in male or female mice. *P < 0.05, **P < 0.01, ***P < 0.001, *vs* control group, one-way ANOVA and Dunnett test, Kruskal-Wallis test. mean ± S.D., n=6~9(male: n=2~4, female: n=4~6).

In order to investigate the effect on secretion of hormones of the HPA and HPG axis in old SAMR1 mice following drug treatment, the hormone levels in the blood, hypothalamus, and hypophysis were detected by radioimmunoassay and ELISA. The results showed that the concentration of CORT(*P* < 0.001, F=5.147) (**Figure 6A**) by donepezil administration, GH (*P* < 0.01) (**Figure 6B**) by melatonin administration in plasma, and TSH (*P* < 0.05, F=3.945) in pituitary by LW administration (**Figure 6D**) were significantly increased compared with the old SAMR1 mice group. Besides, there were an increase trend in GH (**Figure 6B**) and DHEA (**Figure 6C**) secreted by LW administration, as well as the ovarian and sex hormones, including ACTH (**Figure 6E**), LH (**Figure 6F**), and E2 (**Figure 6G**) compared with old SAMR1 mice, but the T and ratio of T/E2 have no significant changes in old female SAMR1 mice (**Figure 6H, I**). Because of the number of male mice in the control group was less than three, we did not analyze the sex hormone in male mice. We found donepezil might further increase the corticosterone levels in aging mice. The data suggest that melatonin promote GH secretion might be beneficial to delaying aging performance, and LW could increase the TSH secretion, which is beneficial to delaying aging as well.

### Correlation Analysis Between Neuroendocrine–Immune Factors and Cognitive Behavior in Old SAMR1 Mice

**Figure 7:**
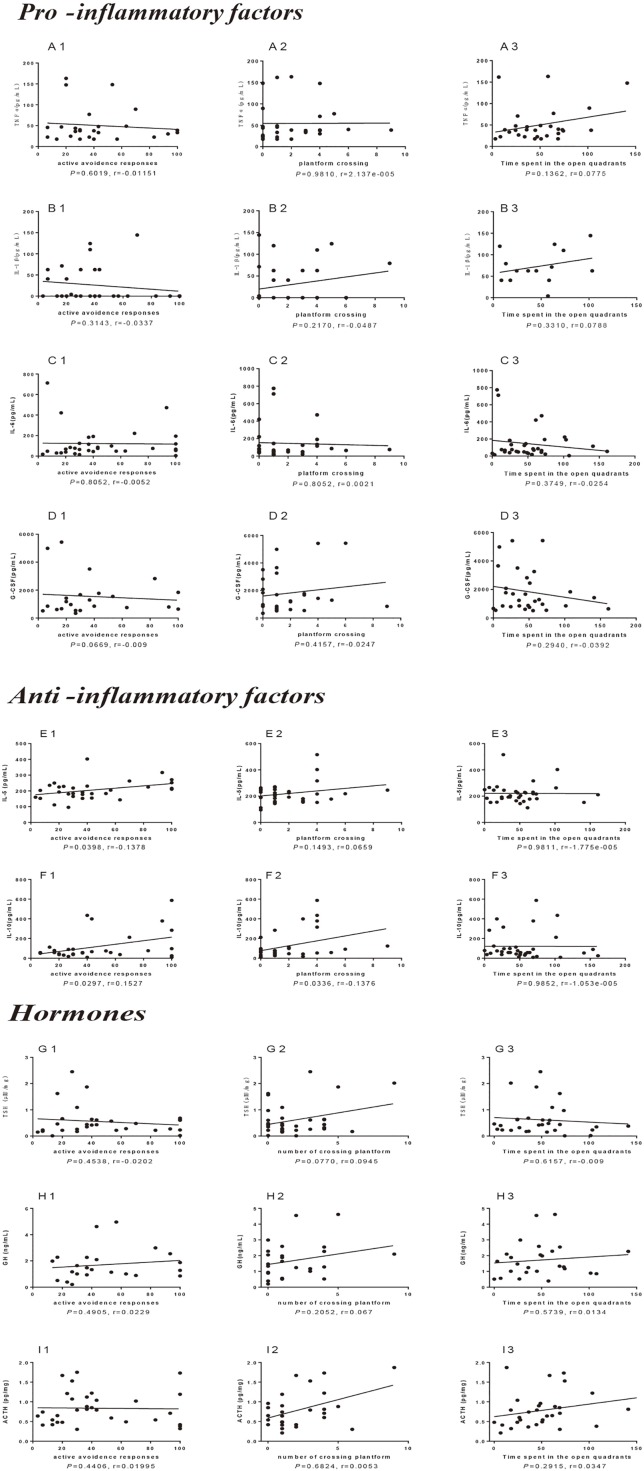
Correlation analysis between neuroendocrine-immune factors and behavior tests in old SAMR1 mice. The pro-inflammatory factors **(A1-3)** TNFα, **(B1-3)** IL-1β, **(C1-3)** IL-6, and **(D1-3)** G-CSF as well as the anti-inflammatory factors **(E1-3)** IL-5 and **(F1-3)** IL-10, hormones **(G1-3)** TSH, **(H1-3)** GH and **(I1-3)** ACTH related with active avoidance response, spatial memory and anxiety behavior of old SAMR1 mice. Pearson correlation analysis, n = 24-33, *P* < 0.05 was considered to be a significant correlation.

To investigate the relationship between neuroendocrine–immune factors and cognitive behavior in old SAMR1 mice, a Pearson correlation analysis was conducted between several inflammatory factors, hormones and the success rate of active avoidance, special memory, and anxiety-like behavior in SAMR1 mice. The results showed that the pro-inflammatory factors level in aged mice had no significant correlation with active avoidance response and spatial learning and memory (**Figure 7A–C**), while there was a negative correlation trend between the levels of G-CSF (**Figures 7D1, D3**) and active avoidance responses and anxiety behavior. The anti-inflammatory cytokines IL-5 (**Figure 7E1**) and IL-10 (**Figure 7F1**) in plasma were positively correlated with active avoidance response, and the level of IL-10 (**Figure 7F2**) in plasma was also significantly positively correlated with special memory of aged mice. For the endocrine factors, the data indicated that TSH (**Figure 7G2**), GH (**Figure 7H2**), and ACTH (**Figure 7I2**) had an obvious positive correlation trend with the special memory of aged mice. These data indicated that the anti-inflammatory factors IL-5 and IL-10 are involved in the cognitive function of aged mice, and this might be an important reason for cognition improvement with LW administration; the level of pituitary hormone secretion might be positively correlated with cognitive function as well. From the above results, we know that administrations of LW and melatonin have a better effect on improving the active response; meanwhile, LW could improve anxiety behavior in aging mice, and LW exhibited an obvious upregulation effect on anti-inflammatory factor IL-4, IL-5, and IL-10. LW may consequently exhibit a better anti-aging effect than other chemical drugs used in this study.

## Discussion

The proposed “Immunosenescence” theory that is the fundamental phenomenon of the immune response referrs to the gradual deterioration of the immune system with aging; inflammation is increased in the absence of infection, and it is also a major factor simultaneously accompanied by cognitive decline in the elderly (Gruver et al., 2007). The hippocampus is a particularly interesting brain region that is known to be associated with learning and memory function as well as mood regulation (Kirasic et al., 1996; Jeste et al., 2010; Salthouse, 2010b). It has been reported that the level of pro-inflammatory cytokines in plasma is generally increased in the elderly (Di Benedetto et al., 2017), which is related to the decrease of hormone levels and chronic inflammation occurence in the aging process (Sherwin, 2002; Matousek and Sherwin, 2010; O’Shea et al., 2016), and the responsiveness, spatial ability test, memory, and information processing decreased significantly in old people as well as mice with aging. Therefore, regulating the balance of neuroendocrine–immune system dysfunction during aging is a therapeutic strategy to alleviate cognitive decline caused by aging. The acetylcholinesterase inhibitor donepezil and the NMDA antagonist memantine are the main drugs for AD treatment, but whether they have effects on the normal aging-related cognitive decline and immune and endocrine system are still unclear. LW has been proven to have beneficial effects on cognitive impairment and neuroprotection, such as murine models of AD (Sangha et al., 2012), PD (Tseng et al., 2014), and D-galactose-induced aging (Zhang et al., 2011). According to the literature, we selected 10 mg/kg memantine (Rosi et al., 2006) and 1 mg/kg donepezil (Solomon and Taghogho, 2017), which have a certain improving effect on cognitive impairment in an AD murine model, and the anti-aging agent melatonin at 10 mg/kg (Yang and Li, 2000) and 10g/kg LW, which is equivalent to the clinical dose. In this study, we found that long-term administration of LW, memantine, and donepezil significantly enhanced the active avoidance responses in aging SAMR1 mice, and the melatonin and donepezil group did not exhibit good performances in the test task, while donepezil could improve spatial learning ability, and LW and melatonin showed a tendency to improve spatial memory of aging mice. Meanwhile, the mice treated with LW showed better fur gloss than others. Anxiety is related to episodic memory, and had a negative impact on item memory and source memory of the elderly (Küçük et al., 2008); evidence showed that old mice spent a longer time in closed arms than younger mice, and older females were more anxious than males and young females (Frick et al., 2000). Our results showed that long-term administration of LW significantly increased the exploration time and frequency in open areas of aging mice, which suggests that LW is beneficial to the improvement of emotional changes in aging mice. Besides, we found an intriguing effect of memantine that was different from the active avoidance response: the spatial learning and memory performance of memantine-treated mice were even worse than the control group. A study demonstrated that a strong disruption of cognitive flexibility in the Morris water maze task following memantine administration in mice (Saab et al., 2011), and a finding also showed that high-plasma memantine dosage was associated with confusion in human patients (Johannes et al., 2007); thus, unusual detrimental effects of memantine require deeper study in the future.

T-cell changes affect brain function (Gemechu and Bentivoglio, 2012; Ks et al., 2014); T-cell deficiency leads to cognitive decline, and it also decreases the ability of T cell to assist B-cell activation and antibody production with aging (Saurwein-Teissl et al., 2002; Frasca et al., 2008). The reduced CD4^+^T cells are defined as an important indicator of immunosenescence (Kovaiou and Grubeck-Loebenstein, 2006; Arsun et al., 2013). CD28 is an important costimulatory molecule that participates in the proliferation and activation of T cells, and it also stimulates the production of many cytokines, such as IL-2, TNFα, and INF-γ, which are important molecules for activating T cells and macrophages (Saurwein-Teissl et al., 2002). CD8^+^CD28^+^T cells and CD4^+^CD28^+^T cells decreased with aging (Vallejo et al., 1998; Broux et al., 2012), and the accumulation of CD28^-^T cells is an obvious marker of aging (Lang et al., 2010). Melatonin exhibited immunoregulatory properties, and, in this study, we found that it could also increase the ratio of CD8^+^ cell and CD8^+^CD28^+^cell subsets and promote the GH secretion in aging mice. We did not, however, observe significant changes in cytokines, and this might due to the use of melatonin as an immune modulator with buffering properties, allowing for immune stimulation in response to low-grade challenges but mitigating severe damage in high-grade inflammation. Furthermore, the accumulation of CD4^+^CD25^+^T cells by aging was capable of decreasing the cytotoxic activity of CD8^+^T cells and production of IL-2 (Trzonkowski et al., 2006). Evidence also showed that memantine relieves the development of arthritis by regulating T-cell functions (Lindblad et al., 2012). Our findings demonstrated that LW administration remarkably increased the CD4^+^T and CD4^+^CD28^+^T cells in the periphery, as did donepezil and memantine. Melatonin significantly downregulated the CD4^+^CD25^+^ T cells and upregulated the CD8^+^CD28^+^T cells, as did memantine. The data suggested that LW, donepezil, and memantine could increase the response and proliferation of CD4^+^T cells, and melatonin has a regulatory effect on T-regulatory (Treg) cells; the regulation of T cells may be an important aspect of alleviating chronic inflammation in the process of aging.

As major mediators of the immune response, cytokines affect many different biological functions, and maintaining the balance of circulating cytokines is thus critical for delaying the aging process. A large amount of data indicated that pro-inflammatory factors, IL-1β, IL-6, and TNFα, increase with aging (Morley and Baumgartner, 2004; Doria and Frasca, 2010; Alvarezrodríguez and Al, 2012), and our previous study also showed that IL-1β, IL-6, TNFα, and G-CSF increased and IL-4, IL-5, and IL-10 significantly decreased in aging SAMR1 mice (J. Wang et al., 2016). Studies reported that IL-4 and IL-5 as reliability age-related markers might have correlated with behavior (Scheinert et al., 2015). Moreover, some serum cytokine concentrations were related to non-spatial cognitive performances, and a higher IL-6 level was associated with better non-spatial learning in aging rats. It is therefore difficult to have a clear understanding of the harmful or beneficial effects of the pro-inflammatory factors throughout life span. Donepezil has been reported to be able to regulate the secretion of MCP-1 and IL-4, and it might have an effect on modulating transformation of Th0/Th2 cytokines (Marcella et al., 2006). This may be an explanation for donepezil’s ability to delay cognitive decline. A correlation analysis indicated that IL-5 and IL-10 are positive corelated with cognitive improvement; LW decreased the level of TNF-α in plasma, and it also increased the anti-inflammatory factors IL-4, IL-5, and IL-10 in the plasma and spleen, promoted Th2-cell immune responses, and maybe also be associated with delaying age-related cognitive decline. By contrast, donepezil and memantine could reduce the circulating inflammatory factor TNF-α, but the integral regulation of donepezil, memantine, and melatonin on cytokines was weaker than LW. The pro-inflammatory factors TNFα and IL-6 (male mice) were increased in the spleen by LW treatment, which seem to be a paradoxical effect. The spleen is the largest immune organ of our body, and it contains many kinds of active immune cells and cytokines. Aging could lead to disorders of spleen function, which further the course of the decline of the body’s immune function. Most of TCM is occupied with dual-directional immunoregulation, which could make high or low immune responses return to a normal level. LW is a classical prescription for nourishing Shen Yin. LW could improve lymphocyte activity, especially that of T cells, promote the secretion of cytokines that may enhance the immune function of the spleen in aging mice, while the cytokines in plasma are more involved in the occurrence of inflammation, and the anti-inflammatory role of LW might be in a dominant position in the blood. This action may be one of the features of dual-directional regulatory effect of TCM. Thus, compared with chemical drugs, LW has the advantage of systematic regulation based on a holistic view for diseases involving complex factors.

Furthermore, the hypothalamus-pituitary-gonad (HPG) axis plays an important role in the neuroendocrine immunomodulation network; increased corticosterone in old people is a recognized physiological change, and the levels of GH and DHEA were decreased. Additionally, hormones affect T-cell differentiation (Murphy et al., 1992). In this study, the results showed that long-term administration of LW and melatonin increased GH and DHEA levels, and the TSH increased with LW treatment, but the CORT level increased significantly after donepezil administration, which deserves our attention. According to the literature, the relationship between sex hormones and cognitive function is complex. Experimental evidence showed that androgens are involved in regulating gender differences in brain structure and cognitive function (Hogervorst et al., 2004). Testosterone exerts neuroprotective effects, and may even simultaneously reduce neuronal apoptosis, increase neuronal activity, and prevent the formation of amyloid plaques and tangles under an optimal level (Holland et al., 2011). Meanwhile, serum levels of estradiol and testosterone exhibit different performances in different cognitive domains in healthy elderly men and women (Hogervorst et al., 2004), such as higher estradiol/testosterone levels associated with better verbal memory in women (Wolf and Kirschbaum, 2002), and adding testosterone to high-dose estrogen substitutes can protect postmenopausal women’s cognitive function (Wisniewski et al., 2002). We found that the levels of ACTH and LH in aging mice displayed an upward trend with melatonin and LW treatment, and the ratio of T/E2 also have upward trend with LW treatment, especially in aging male mice. Sex hormones can enhance or weaken the immune response by regulating the signal system of T-cell receptors, altering the expression of activating molecules on the surface of T cells and antigen-presenting cells, influencing the transcription and translation of cytokine genes (Yron et al., 1991; Krzych et al., 1981). A well-balanced ratio between estrogen and testosterone levels may be helpful for good cognition, and the effect of these drugs on hormone secretion may be helpful to further develop a more comprehensive understanding of cognitive impairment induced by aging or neurodegenerative disease.

In conclusion, our present study compared the effects of modern agents donepezil, memantine, and melatonin and a classical TCM, LW, on cognition and neuroendocrine–immune responses of aged SAMR1mice. We found that LW and melatonin could extend the survival days of old mice and gave them glossier fur; the anxiety behavior was significantly improved by use of memantine, melatonin, and LW treatment, active avoidance responses significantly improved by use of LW, donepezil, and memantine, and the spatial learning ability was significantly improved by donepezil. LW and melatonin, however, exhibited beneficial effects in relation to the spatial memory of old mice. For the immune system, donepezil, memantine, and LW significantly increased the proliferation of active T cells, and increased the CD4^+^CD28^+^ subsets, which is beneficial to anti-aging, while the melatonin was beneficial to CD8^+^ T-cell subset responses and B cells. Meanwhile, donepezil and memantine significantly decreased the pro-inflammatory factor TNF-α in plasma, and IL-5 and IL-10 showed a positive relationship with cognition. Treatment with just LW significantly increased the levels of anti-inflammatory factor IL-5 and IL-10. For endocrine factors, LW as more beneficial to the regulation of HPG axis hormones than others. Overall, the results indicate that LW improved the cognitive decline in aging mice might be associated with modulation of the active T cells and HPG axis hormones and increased anti-inflammatory factors produced by the Th2 cells. Meanwhile, we found that donepezil and memantine have advantages in regulating adaptive immunity, while melatonin has advantages in the regulation of B cells and pituitary hormones, and LW has a more comprehensive regulatory effect on neuroendocrine–immune function compared with others. LW might be a potential therapeutic strategy for anti-aging-related syndromes, and it can also provide a value in terms of medication guidance for drug combinations or treatment in clinic.

## Data Availability Statement

All datasets generated for this study are included in the article/**Supplementary Material**.

## Ethics Statement

The animal study was reviewed and approved by The animal received humane care according to the National Institutes of Health (USA) guidelines, approved by the Institute of Animal Care and Use Committee (IACUC) of the National Beijing Center for Drug Safety Evaluation and Research (NBCDSER) (No. 2018-030).

## Author Contributions

XC, WZ, and YZ conceived the study, participated in its design and coordination, and helped to draft the manuscript. JZ carried out all experiments and wrote and revised the manuscript. XC participated in the design of the study and wrote and revised the manuscript. XZ and WZ participated in the flow cytometric analysis, evaluation of the degree of senescence, and nest building. JW participated in the evaluation of the degree of senescence and nest building. All authors read and approved the final manuscript.

## Funding

This research was funded by the National Natural Science Foundation of China (81473191) and the national key research and development program (2016YFC1306300).

## Conflict of Interest

The authors declare that the research was conducted in the absence of any commercial or financial relationships that could be construed as a potential conflict of interest.
