# Supplementary material for: Comparison of Donepezil, Memantine, Melatonin, and Liuwei Dihuang Decoction on Behavioral and Immune Endocrine Responses of Aged Senescence-Accelerated Mouse Resistant 1 Mice
Source: Front Pharmacol. 2020 Apr 28;11:350. doi: 10.3389/fphar.2020.00350 (PMC7241684; doi:10.3389/fphar.2020.00350)
Supplement: Supplementary file 1 [file DataSheet_1.docx]

**Temporal order memory test**

The exploration apparatus is an open-topped arena (50×50×50 cm) made of black plastic. The A object was square pyramid and B object was conical flask filled ink that were too heavy for the animal to displace. This task comprised two sample phases and one test phase. Before this task, each mouse was placed into apparatus for 20 min allowed to freely explore for 2 days, then returned to their home cage. In each learning phase, the mice were allowed to explore two copies of an identical object for 15 min, with a delay between the learning phase1 and 2 of 1 h. The test phase was given 3 h after learning phase 2, a copy of the object A from phase 1 and a copy of object B from phase 2 were used, and the positions of the objects in the test not changed, the mice were allowed to explore for 4 min. If temporal order memory is intact, the mice will spend more time exploring the object A. The discrimination ratio was calculated as the time spent by each mouse exploring the object A divided by the total time spent exploring both objects in the test period. Using an overhead camera and video recorder to record the mouse’s behavior.

The results showed that the control group showed similar exploration time in object A and B, which meaning the temporal order memory was disorder in old SAMR1 mice, while the memantine, melatonin and LW group mice spent more time for exploring object A than object B, (Figure S1), this suggests that the mice have a better memory of the later object, so memantine, melatonin and LW might have tendency in improve the temporal order memory in OLD SAMR1 mice.

B

A

D

C

**FIGURE S1 Effects of donepezil, memantine, melatonin and LW on discrimination ration of object in old SAMR1 mouse**. The exploration time of A object (A) and exploration time of B object (B) in learning phase, (C) the exploration time in test phase of A and B object, (D) the discrimination ratio of A and B object. One-way ANOVA and Dunnett test，mean±S.D. n=6~9


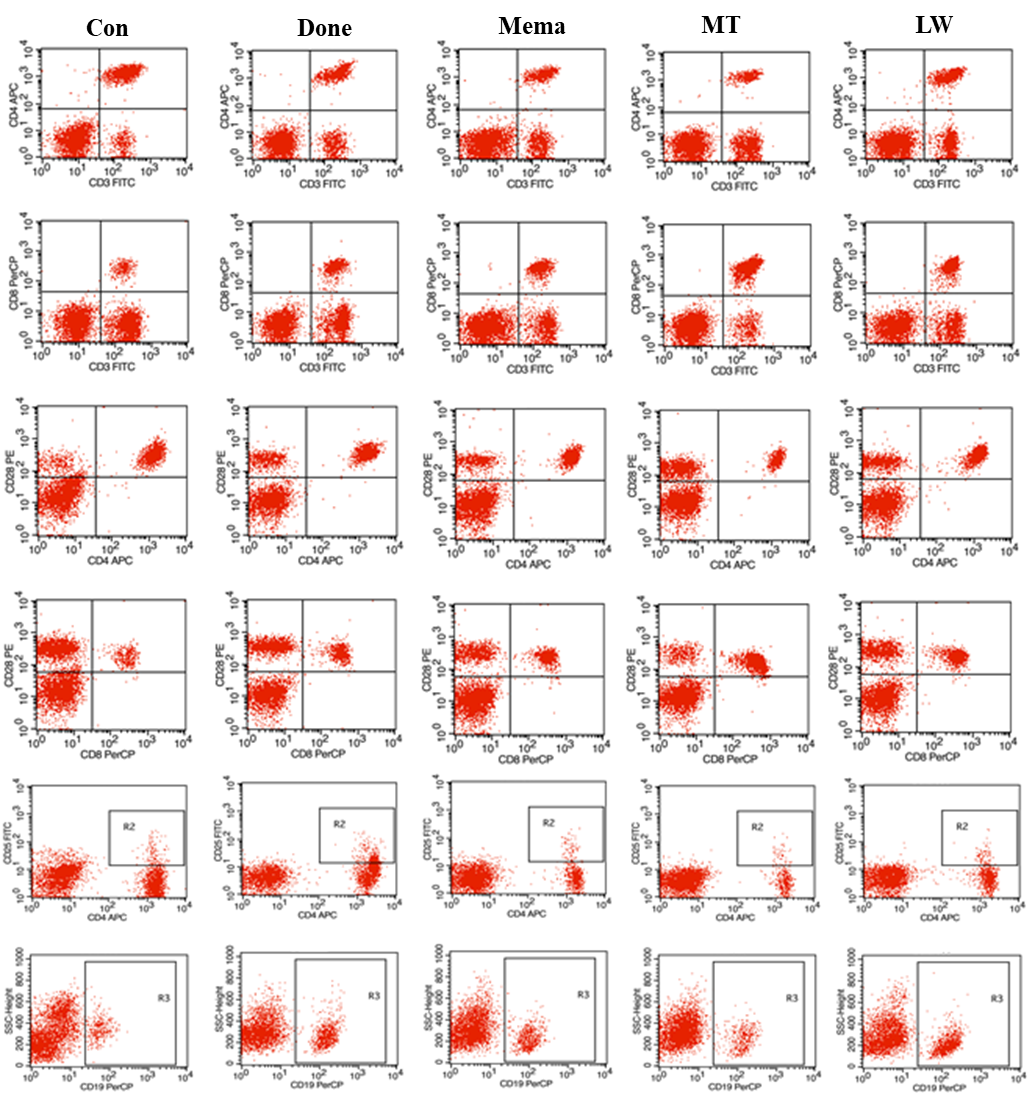


**FIGURE S2 Effects of donepezil, memantine, melatonin and LW on lymphocyte subsets in blood of old SAMR1 mice. (A)** CD19^+^ B cells, **(B)** CD3^+^ CD4^+^ T cells, **(C)** CD3^+^ CD8^+^ T cells, **(D)** CD4^+^ CD25^+^ T cells, **(E)** CD4^+^ CD28^+^ T cells, **(F)** CD8^+^ CD28^+^ T cells quantified by flow cytometry. **P*<0.05, ***P*<0.01, ****P*<0.001, *vs* control, one-way ANOVA and Dunnett test. mean±S.D, n=6~9.
